# On the History, Presence, and Future of Optics Manufacturing

**DOI:** 10.3390/mi12060675

**Published:** 2021-06-09

**Authors:** Christoph Gerhard

**Affiliations:** Faculty of Engineering and Health, University of Applied Sciences and Arts, Von-Ossietzky-Straße 99, 37085 Göttingen, Germany; christoph.gerhard@hawk.de; Tel.: +49-(0)551-3705-220

In 1850, Austen Henry Layard discovered an approximately 3000-year-old, simple optical lens in Nimrud, Northern Iraq—the Nimrud lens, aka the Layard lens [1,2]. About 20 years later, Heinrich Schliemann found several lenses during excavations in the famous archeological site of Troy, Turkey [3]. It is also known that the Vikings used aspheric lenses [4]—the so-called Visby lenses, found in Fröjel on the Swedish island of Gotland in 2002, that were buried around 1050 AD, and thus produced more than 1000 years ago.

Based on these discoveries and evidences, it can be stated out that the production of imaging optical elements has been performed for more than 5000 years. In modern times, optics manufacturing began in the early 17th century, when the spectacle-maker Hans Lipperhey patented his famous telescope. It is said that, to improve the imaging quality of such telescopes, Galileo Galilei acquired contemporary lens making skills. Optics manufacturing was a purely manual work until the invention of the first grinding machines by Joseph von Fraunhofer and Johann Heinrich August Dunker, at the beginning of the 19th century. This achievement allowed for large-scale industrial production, which was brought to perfection by the cooperation of Carl Zeiss, Ernst Abbe and Otto Schott in the late 19th century—a cooperation where not only optics manufacturing, but also the production of optical glasses with well-defined properties, was covered [5]. In the 1980s and 1990s, new computer-aided and controlled approaches, as well as standardized polishing processes, opened new dimensions in the production of optics for mass markets.

The growing demand for optical components of high quality and performance, for example, for the realization of UV optics or high-power laser devices, requires the development of novel manufacturing approaches on the one hand, and a better understanding of tool–glass interactions on the other hand. At present, glasses—or other optical media such as sapphire—are polished on the atomic scale to address the tightest requirements regarding surface roughness. Moreover, stable methods with high repeatability are needed for the aspherization or sub-aperture polishing of freeform optics. Finally, the reduction in surface or subsurface damage and contamination of polished surfaces has become an important issue [6].

Some challenges in modern optics manufacturing can be successfully accomplished by classical methods, tools and operating materials. However, the diversity of manufacturing techniques has increased significantly in the recent decades; novel approaches such as magneto-rheological finishing [7], plasma etching [8], ion beam etching, fluid jet polishing [9], or laser-based structuring and smoothing have been introduced to provide solutions for special production tasks. It can thus be summarized that, even though optics manufacturing is a handcraft with a millennia-old history and tradition, its adaption and development is not yet finished, and will almost certainly remain an issue in the future.
